# Polyamines under Abiotic Stress: Metabolic Crossroads and Hormonal Crosstalks in Plants

**DOI:** 10.3390/metabo2030516

**Published:** 2012-08-20

**Authors:** Marta Bitrián, Xavier Zarza, Teresa Altabella, Antonio F. Tiburcio, Rubén Alcázar

**Affiliations:** Unit of Plant Physiology. Department of Natural Products and Plant Biology, Faculty of Pharmacy, University of Barcelona, Diagonal, 643, 08028 Barcelona, Spain

**Keywords:** polyamines, stress, metabolism, SAM, GABA, proline, ABA

## Abstract

Polyamines are essential compounds for cell survival and have key roles in plant stress protection. Current evidence points to the occurrence of intricate cross-talks between polyamines, stress hormones and other metabolic pathways required for their function. In this review we integrate the polyamine metabolic pathway in the context of its immediate metabolic network which is required to understand the multiple ways by which polyamines can maintain their homeostasis and participate in plant stress responses.

## 1. Introduction

Abiotic stresses such as cold/freezing, salinity, heat and drought represent serious threats to agriculture. Climatic change is predicted to increase global temperature, alter precipitation patterns and intensify drought, increasing the need to grow crops in saline soil [1,2]. Plants, which are sessile organisms, have evolved metabolic and hormonal pathways to cope with environmental challenges. The study of this natural evolution on stress responsiveness is providing new leads to crop protection. In recent years, genetic and genomic approaches have revealed complex metabolic and hormonal networks which are coordinated to provide an optimal response to stress conditions. Efforts have created network models of stress and hormone regulatory pathways, as well as the definition of frameworks of co-regulated target genes of abiotic stress response pathways e.g. [3]. It is generally recognized that the study of stress responses must be integrated in the context of a multiple response involving different plant metabolites and hormones rather than in isolated pathways. Global transcriptional and metabolomic approaches very frequently recognize the participation of polyamines (PAs) in a large number of abiotic and biotic stresses. In this review we synthesize what is known about direct interactions of PAs with other metabolic pathways during the stress response. We also discuss the dependence of PA responses on the stress hormone abscisic acid (ABA) during drought.

## 2. Polyamine Biosynthesis and Its Interaction with Other Metabolic Pathways

**Figure 1:**
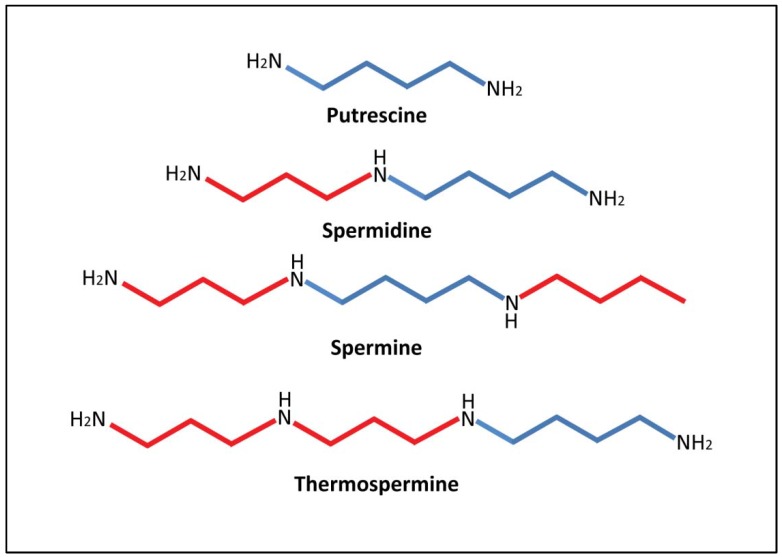
Chemical structure of putrescine, spermidine, spermine and thermospermine. Aminopropyl moieties (in red) are added to the four carbon linear chain skeleton of putrescine (in blue) to produce spermidine, spermine and thermospermine.

PAs are polycationic compounds of low molecular weight which are present in most living organisms [4]. The diamine putrescine (Put), triamine spermidine (Spd), tetramines spermine (Spm) and thermospermine (tSpm) can be found in free (Figure 1) and conjugated forms. The homeostasis of PAs in the cell is mainly achieved through the regulation of its biosynthesis and catabolism. However, polyamine (PA) conjugation in form of hydroxycinnamic acid amides such as coumaroylputrescine, feruloylputrescine, dicoumaroylspermidine, diferuloylspermidine or diferuloylspermine among others, significantly contributes to the regulation of free PA levels in plants [5]. Through the regulation of PA biosynthesis, catabolism and conjugation, the free levels of PAs are tightly regulated and generally only oscillate in response to environmental insults (e.g. stress) or during the transition between different developmental stages (e.g. flowering). In the following sections we integrate the PA pathway in the context of a wider metabolic network and discuss current evidences supporting a role for PAs in drought protection.

### 2.1. Biosynthesis of Putrescine. Interactions with Proline, Urea, NO and Alkaloid Biosynthesis

**Figure 2:**
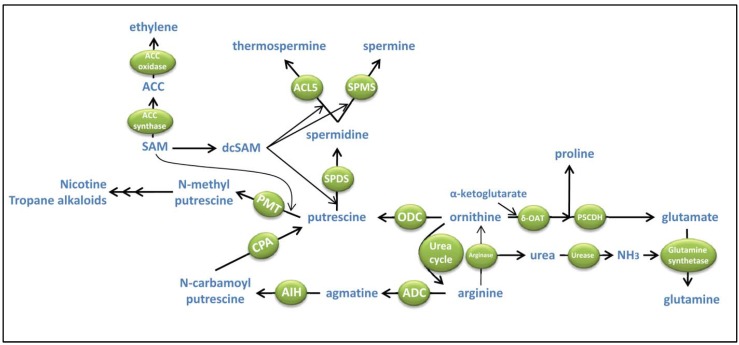
Biosynthesis of polyamines and connections with other metabolic pathways. ACC, aminocyclopropane carboxylic acid; ACL5, ACAULIS5 - thermospermine synthase; ADC, arginine decarboxlase; AIH, agmatine iminohydrolase; CPA, N-carbamoylputrescine amidohydrolase; dcSAM, decarboxylated SAM; δ-OAT, δ-ornithine aminotransferase; ODC, ornithine decarboxylase; P5CDH, pyrroline 5-carboxylate dehydrogenase; PMT, Putrescine N-methyltransferase; SAM, S-adenosylmethionine; SAMDC, S-adenosylmethionine decarboxylase; SPDS, spermidine synthase; SMPS, spermine synthase.

The first PA synthesized in the PA-bioynthetic pathway is Put (Figure 2). This PA can be derived from the decarboxylation of the amino acid ornithine through an enzymatic reaction catalyzed by ornithine decarboxylase (ODC, EC 4.1.1.17; Figure 2). Whereas this enzymatic step is generally recognized to be present in all living organisms, evidences are found that the model species *Arabidopsis thaliana* does not have a functional ODC pathway [6]. In addition, some of the regulatory mechanisms involved in the regulation of ODC activity in mammals, such as the ubiquitin-independent proteasome degradation of ODC by a PA-induced protein called ‘antizyme’, are not found in plants [7,8]. Hence, it is likely that the ODC pathway, as well as the regulation of ODC activity, has evolved differently in plants compared to mammals, leading to an eventual loss of this pathway in some plant species studied. Loss of ODC pathway might be due to the evolution of an alternative route to Put biosynthesis present in plants and bacteria [4], the arginine decarboxylase (ADC) pathway, which uses arginine as substrate for Put biosynthesis (Figure 2).

Arginine to put conversion requires three consecutive enzymatic reactions (Figure 2). The first step is the enzymatic decarboxylation of arginine by ADC (EC. 4.1.1.19). The product of this enzymatic reaction is agmatine, which serves as substrate for agmatine iminohydrolases (AIH, EC. 3.4.3.12) to produce N-carbamoyl-Put. The final step in the ADC pathway is the hydrolysis of N-carbamoyl-Put to Put by N-carbamoyl-Put amidohydrolases (CPA, EC 3.5.1.53). Despite the need of three consecutive enzymatic reactions for the arginine to Put conversion, evidences indicate that the limiting enzyme in Put biosynthesis is ADC [4,9]. Hence, manipulation of Put biosynthesis in plants can be achieved by the genetic manipulation of ADC activity such as over-expression of ADC encoding genes. In this way, plants accumulating high levels of endogenous Put have been generated and showed enhanced resistance to drought and freezing stresses [4,9,10]. Except for few cases [9], increases in Put levels achieved by overexpression of *ADC* are moderate which suggests the occurrence of post-transcriptional regulation of *ADC*, transformation of Put to higher PAs (e.g. Spd, Spm and tSpm), catabolism/conjugation, among others mechanisms to maintain Put homeostasis.

Even though ADC and ODC use different substrates, both pathways for Put biosynthesis are connected by arginase, an enzyme that converts arginine to ornithine. *Arabidopsis thaliana* contains two genes coding for arginase (*ARGAH1* and *ARGAH2*) which can complement the yeast arginase deficient (*car1*) mutant [11]. The expression of *ARGAH1* is restricted to pollen whereas *ARGAH2* expression is induced in leaves by treatment with methyl jasmonate [12]. Flores et al. [13] also reported *ARGAH1* and *ARGAH2* expression in expanded cotyledons and root vasculature of young seedlings, especially in response to the synthetic auxin NAA. ARGAH2 is a major contributor to arginase activity in seedlings and both ARGAH1 and ARGAH2 are localized to the mitochondrial matrix [13]. Interestingly, over-expression of *ARGAH2* in *Arabidopsis thaliana* leads to enhanced resistance to *Botrytis cinerea*, thus suggesting a role for arginase in the biotic stress responses to necrotrophic pathogens at least in this species [14]. 

The hydrolisis of arginine to ornithine is also a source of urea [15] (Figure 2). The ornithine formed by arginase activity can be used for PA biosynthesis in plants provided with an ODC pathway. Alternatively, ornithine can be converted again into arginine through the urea cycle. Another metabolic pathway for ornithine is its catabolism through the activity of ornithine-δ-aminotransferases (δ-OAT, Figure 2). In this enzymatic reaction, the side chain amino group of ornithine is transferred to α-ketoglutarate to generate glutamate and pyrroline 5-carboxylate. The latter compound can be oxidized to glutamate by pyrroline-5-carboxylate dehydrogenase (P5CDH) or alternatively, be reduced by NADPH to generate proline (Figure 2). However, it is still controversial the contribution of δ-OAT to proline levels since *oat* mutants in *Arabidopsis thaliana* do not show altered proline levels under stress [16]. Therefore, it is more likely that the final product from δ-OAT activity is glutamate rather than proline. The urea released by arginase activity is exported to the cytosol and hydrolized by cytosolic ureases to ammonia (Figure 2). Ammonia is then re-assimilated by cytosolic glutamine synthetases to glutamine using the glutamate derived from δ-OAT catabolism (Figure 2). 

Overexpression of *ARGAH2* in *Arabidopsis thaliana* has been shown to lead to increased arginase activity and reduced arginine levels which did not result in lower PA levels [14]. This might be the consequence of the reallocation of free and conjugated PA forms, inhibition of PA catabolism, its transport or other mechanistic processes by which plants maintain PA levels under strict control. The requirement of a minimum PA pool for cell survival likely underlies its tight homeostasis regulation [4]. Interestingly, even though δ-OAT activity does not necessarily lead to increased proline levels [16], overexpression of *ARGAH2* lead to increased proline in some lines [14]. Further genetic analyses would be required to better define the metabolic interaction between arginine and proline mediated by δ-OAT.

In animal cells, arginine is also susbtrate for nitric oxide synthases (NOS) which produce nitric oxide (NO). Nitric oxide plays important roles in the regulation of stomatal movements in response to ABA and is an integral part in many defense and developmental pathways. Although the identification of a NOS orthologue gene in plants that would link arginine to NO biosynthesis is missing [17,18] some reports evidence that PAs, and particularly Spm, induce a rapid NO production without lag [19]. Increased NO observed in arginase mutants *argah1-1* and *argah2-1* has been suggested to derive from an increased availability of arginine for PA biosynthesis [13]. The source for NO production induced by PAs still remains to be elucidated [20]. 

In the crossroad of PAs with other metabolic pathways, the interaction of Put with secondary metabolism is well established in some plants. The enzyme Putrescine N-methyltransferase (PMT, Figure 2) catalyzes the methylation of Put using SAM as methyl donor. The product of this enzymatic reaction, N-methyl putrescine, is required for the synthesis of nicotine, tropane and nortropane alkaloids in Solanaceae and Convolvulaceae plants [21].

### 2.2. Biosynthesis of Spermidine, Spermine and Thermospermine. Interactions with S-adenosylmethionine (SAM) and ethylene

Higher molecular weight PAs spermidine and spermine are synthesized by the sequential addition of aminopropyl moieties to the four carbon linear chain skeleton of Put (Figure 1) through enzymatic reactions catalyzed by Spd and Spm synthases, respectively (SPDS, EC 2.5.1.16 and SPMS, EC 2.5.1.22; Figure 2). 

The donor of aminopropyl groups is decarboxylated S-adenosyl methionine (dcSAM) which is synthesized from decarboxylation of S-adenosyl methionine (SAM) by SAM decarboxylases (SAMDC, EC 4.1.1.50; Figure 2). The availability of dcSAM limits the biosynthesis of Spd and Spm [22]. This makes SAMDC one of the major regulators of the PA biosynthetic pathway. Furthermore, SAMDC competes for SAM, which is used as universal methyl donor in many enzymatic reactions involving *O-*, *N-* and *C-*methyltransferases in primary and secondary metabolism [23]. dcSAM can only be used for PA biosynthesis and therefore, the regulation of SAMDC activity is likely to be relevant in the context of a complex metabolic network. Indeed, the transcriptional regulation and biochemical properties of SAMDCs are tightly regulated and have been extensively studied [24]. As anticipated, SAM is precursor of other metabolites in plants. Relevant for crop stress protection against nutritional deficiency is the requirement of SAM for the synthesis of nicotianamine by nicotianamine synthase [25]. Nicotianamine is a strong chelator of iron and in graminaceous plants is precursor of phytosiderophores required for iron uptake from soil [26]. Nevertheless, a potential interaction between PA biosynthesis and iron uptake efficiency is unknown. SAM is also substrate for ethylene biosynthesis in higher plants (Figure 2). The enzyme 1-aminocyclopropane-1-carboxylate (ACC) synthase converts SAM to ACC, which is oxidized by ACC oxidase to ethylene [27,28]. Hence, PAs and ethylene could act in an antagonistic manner competing for the common substrate SAM. Whereas ethylene would contribute to senescence and fruit ripening, PAs would favor growth and inhibit senescence [29]. Indeed, anti-senescent properties of PAs are well documented [30]. Some of the PA-related antisenescence effects might be due to the PA-mediated inhibition of ethylene biosynthesis. Indeed, examples are found in literature that the exogenous application of PAs inhibits ethylene biosynthesis [31,32,33]. In tomato, PAs have been shown to inhibit the induction of ACC synthase by wounding [34]. In carnation flowers [35] the blockage of SAM to ACC conversion was translated in an increase of Spm levels whereas impairment of PA biosynthesis promoted ethylene pathway and senescence. However, most of these analyses are based on exogenous applications of PAs, ethylene and their inhibitors which have some intrinsic limitations. Furthermore, the antagonistic effect between PAs and ethylene is not always observable. Mehta et al. [36] reported that tomato plants transformed with yeast *SAMDC* under the control of the ripening specific E8 promoter synthesized more ethylene and PAs concomitantly during fruit ripening, thus evidencing the absence for SAM competition in this system. Initial studies already pointed to other mechanisms rather than a mere metabolic competition for SAM involved in the PA-ethylene antagonism [31,32,33] although scarce attention has been taken in the recent years to the PA-ethylene crosstalk. In a recent work, overexpression of yeast *Spermidine Synthase* in tomato has been shown to produce plants susceptible to the fungal necrotroph *Botritis cynerea* concomitantly to a repression of ethylene biosynthesis and signaling, thus providing support to the view that PA-ethylene cross-regulation occurs and has important implications for defense [37]. Further analyses would be required to identify the interacting nodes in this PA-ethylene cross-regulation.

### 2.3. Put to Spm Canalization and Its Contribution to Stress Protection

Higher PAs, Spd and Spm have been shown to significantly contribute to abiotic stress protection although their specific mechanism(s) of action remain to be elucidated. Some of the genetic evidences have been obtained from the analysis of loss-of-function mutations in *Arabidopsis thaliana*. This species carries two genes coding for Spd Synthase (*SPDS1* and *SPDS2*), and one Spm Synthase (*SPMS*) [4]. Interestingly, *Arabidopsis thaliana* also contains one gene (*ACAULIS5*, *ACL5*) coding for thermospermine synthase, an enzyme involved in the synthesis of the Spm isomer thermospermine (tSpm) [38]. Regardless of its low levels, tSpm is required for proper vascular tissue development and stem elongation, phenotypes which are evidenced in the *acl5* mutant [39]. The *spms-1* mutant impared in Spm biosynthesis has no evident morphological alterations and it is viable [40]. The double *spms-1/acl5-1* mutant lacking both Spm and tSpm is also viable and shows an *acl5-1* phenotype [39,40]. However, impairment of Put or Spd biosynthesis leads to embryo lethality [41,42]. Consistent with a protective role of Spm and/or tSpm under abiotic stress, the double *spms-1/acl5-1* mutant has also been reported to exhibit enhanced sensitivity to drought and salinity [43,44]. A potential role for tSpm in biotic stress protection in *Arabidopsis thaliana* has recently been proposed [45].

In a recent report [46], a Put to Spm metabolic canalization in response to drought was revealed in *Arabidopsis thaliana* and the resurrection plant *Craterostigma plantagineum*. In this work, the levels of PAs were analyzed in response to drought in *Arabidopsis thaliana* mutants impaired at different steps of the PA biosynthetic pathway. This approach allowed monitoring the accumulation of PAs during the course of dehydration. In this way, it could be revealed that even though *Arabidopsis thaliana* wild type plants do not accumulate Spd or Spm in response to drought, a Put to Spm canalization occurs which is not translated in Spd or Spm increases. Conversely, the drought tolerant species *Craterostigma plantagineum* showed dramatic increases in Spd and Spm contents that correlated with drought tolerance. Put to Spm metabolic canalization seems to be a conserved mechanism between species, whereas the ability to accumulate high Spd and Spm levels (e.g. by inhibition of its oxidation) may discern between drought tolerant and intolerant species.

## 3. Polyamine Catabolism. Interactions with GABA

**Figure 3:**
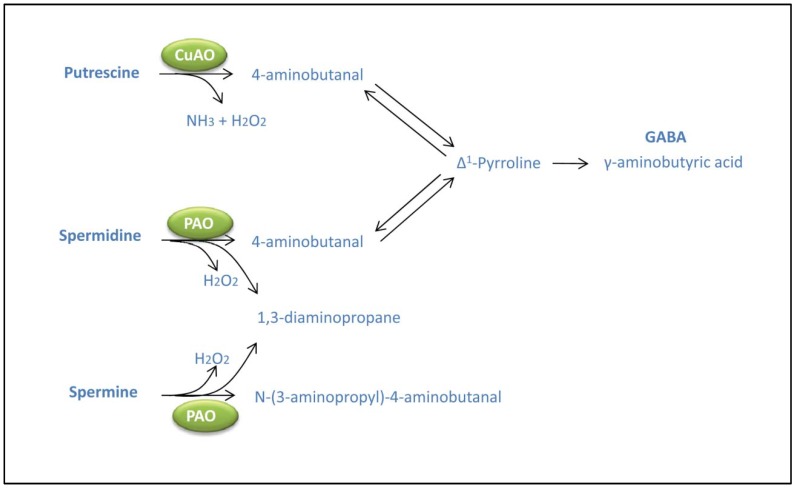
Polyamine catabolism mediated by CuAOs (copper amine oxidases) and PAOs (polyamine oxidases) in plants.

PAs are oxidatively deaminated by the action of amine oxidases. Diamine oxidases (DAOs) are copper-containing amine oxidases (CuAO) which oxidize the diamines Put and cadaverine at the primary amino groups. 4-aminobutanal is produced from the oxidation of Put by DAO activities, concomitantly with the release of ammonia and hydrogen peroxide (Figure 3). Other class of amine oxidases are flavin-containing polyamine oxidases (PAO) involved in the terminal catabolism of Spd and Spm producing 4-aminobutanal or N-(3-aminopropyl)-4-aminobutanal, 1,3-diaminopropane and H_2_O_2_ [47,48] (Figure 3). Another group of PAOs are involved in the back-conversion of Spm to Spd with concomitant production of 3-aminopropanal and H_2_O_2_ [48]. 4-aminobutanal produced in Put catabolism by DAO and terminal catabolism of Spd can be converted to γ-aminobutyric acid (GABA) via Δ1-pyrroline. It is well established the role of GABA as neurotransmitter in animal cells. In plants, increases in GABA levels have been reported in response to different stresses [49] and GABA is suggested to contribute to stress protection through the regulation of cellular pH, acting as osmoregulator or as signaling molecule in plants [50]. However, most of the roles for GABA under stress still need to be defined. In soybean roots exposed to salinity, the degradation of PAs has been associated with increased levels of GABA [51]. Conversely, during the recovery from stress, the levels of GABA are reduced concomitantly to an increase of PAs. [51] These observations suggest that PA catabolism might contribute to increase GABA levels during salinity.

PA oxidation is a source of H_2_O_2 _in the apoplast which can contribute to the defense response against pathogens [47]. Evidences are found that some PAOs participate in the hypersensitive response (HR) in *Nicotiana tabacum* plants resistant to tobacco mosaic virus (TMV). Inhibition of PAO activity by guazatine reduced HR symptoms in TMV infected plants [52]. In a more recent work, H_2_O_2_ derived from PA catabolism has also been associated with non-host induced HR in tobacco, thus leading to the view that PAs could participate in both host and non-host induced HR [53].

## 4. ABA Dependence of PA-Responses under Drought

Abscisic acid (ABA) is a key hormone with important roles in abiotic stress [54]. Many drought-inducible genes are responsive to ABA, but also ABA-independent pathways are activated in response to drought conditions. In order to determine the involvement of ABA in the transcriptional regulation of the PA biosynthetic pathway in response to drought, Alcázar et al. [55] analyzed the expression of PA biosynthesis genes *ADC1*, *ADC2*, *AIH*, *CPA*, *SPDS1*, *SPDS2*, *SPMS*, *ACL5*, *SAMDC1* and *SAMDC2* in *Arabidopsis thaliana* wild type plants and mutants impaired in ABA biosynthesis (*aba2-3*) or signaling (*abi1-1*). *ADC2*, *SPDS1* and *SPMS* genes were among the most responsive ones to drought stress [55]. The increased expression of these three PA biosynthesis genes suggested a potential role for *ADC2*, *SPDS1* and *SPMS* in the drought response. Interestingly, whereas *ADC2* and *SPDS1* expression increased several fold by drought treatment, the expression of their gene paralogs, *ADC1* and *SPDS2*, did not change substantially [55]. These observations are consistent with the acquisition of certain stress-specificity, probably due to divergent evolution of *cis*- regulatory elements in their promoters. Indeed, different *cis* elements are found in the promoters of PA biosynthesis genes [39,56]. ABA-responsive elements (ABRE) or ABRE-related motifs are also found in the promoters of *ADC2*, *SPDS1* and *SPMS* [56], which are highly up-regulated in response to drought [55]. The analysis in *aba2-3* and *abi1-1* mutants showed much more moderate increases in *ADC2*, *SPDS1* and *SPMS* expression [55]. These results evidenced that transcriptional up-regulation of *ADC2*, *SDPS1* and *SPMS* by drought is mediated by ABA. Hence, ABA is an upstream regulator of PA biosynthesis in response to drought. To determine the effect of the transcriptional regulation of PA biosynthesis genes on PA levels, the content of Put, Spd and Spm levels in response to drought were also analyzed. Wild type plants showed a progressive accumulation of Put in response to drought conditions, whereas this accumulation was absent in *aba2-3* and *abi1-1* [55]. Hence, the ABA-dependent up-regulation in *ADC2* expression observed under drought leads to an effective Put accumulation. In a recent work, Alcázar et al. [10] determined a role for *ADC2* overexpression in conferring drought tolerance by transformation of *A. thaliana* plants with the homologous *ADC2* gene under the constitutive CaMV 35s promoter. The different lines analyzed showed contrasting degrees of *ADC2* expression and Put accumulation [9]. Total Put content was between 12- and 2-fold higher than wild type depending on the transgenic line [9,10]. Interestingly, plants that accumulated higher levels of Put were more resistant to drought stress and the enhanced drought tolerance correlated with a reduced stomata aperture and transpiration rate [10]. 

## 5. Future Perspectives

The polyamine field is complex. First because PAs have multiple roles acquired during evolution and it is difficult to disentangle one from the other to study them in isolation. Lethality due to depletion of PA levels is a good example of the intrinsic difficulties of addressing functional questions. To gain a further insight into this topic, we believe that it is necessary to study the interconnections of PA biosynthesis, degradation and conjugation with other metabolic routes in depth and place these compounds in the context of a full metabolic and signaling network. We still need to address where PAs localize, where they are required for their function, how they are transported from sources to sinks (and also which are the sources and the sinks) and more importantly, whether PAs are intermediary compounds in the stress protection or have a role themselves. We believe that a detailed metabolic and signaling analysis addressing these and other fundamental questions is needed to provide a broader view about the roles and mechanisms of PAs during stress.
